# Monitoring calcium-induced epidermal differentiation *in vitro* using multiphoton microscopy

**DOI:** 10.1117/1.JBO.25.7.071205

**Published:** 2020-05-09

**Authors:** Monika Malak, Julie Grantham, Marica B. Ericson

**Affiliations:** a University of Gothenburg, Biomedical Photonics Group, Department of Chemistry and Molecular Biology, Faculty of Science, Gothenburg, Sweden; b University of Gothenburg, Department of Chemistry and Molecular Biology, Faculty of Science, Gothenburg, Sweden

**Keywords:** multiphoton microscopy, epidermal differentiation, keratinocytes, *in vitro* modeling, live imaging, autofluorescence

## Abstract

**Significance:** Research in tissue engineering and *in vitro* organ formation has recently intensified. To assess tissue morphology, the method of choice today is restricted primarily to histology. Thus novel tools are required to enable noninvasive, and preferably label-free, three-dimensional imaging that is more compatible with futuristic organ-on-a-chip models.

**Aim:** We investigate the potential for using multiphoton microscopy (MPM) as a label-free *in vitro* approach to monitor calcium-induced epidermal differentiation.

**Approach:** *In vitro* epidermis was cultured at the air–liquid interface in varying calcium concentrations. Morphology and tissue architecture were investigated using MPM based on visualizing cellular autofluorescence.

**Results:** Distinct morphologies corresponding to epidermal differentiation were observed. In addition, ${\mathrm{Ca}}^{2+}$-induced effects could be distinguished based on the architectural differences in stratification in the tissue cultures.

**Conclusions:** Our study shows that MPM based on cellular autofluorescence enables visualization of ${\mathrm{Ca}}^{2+}$-induced differentiation in epidermal skin models *in vitro*. The technique has potential to be further adapted as a noninvasive, label-free, and real-time tool to monitor tissue regeneration and organ formation *in vitro*.

## 1 Introduction

Current trends in tissue engineering aim for the development of organ-on-a-chip models, potentially enabling personalized drug delivery.^1^ Organ-on-a-chip modeling allows for a three-dimensional (3-D) cell culture in microfluidic devices, mimicking the organotypic environment in health and disease.^2^ This type of advanced tissue engineering is expected to facilitate a shift in science toward more reliable human-oriented organ modeling; however, tools enabling fast, 3-D, and high-resolution investigation of tissue morphology and architecture in real time are lacking. Histology, presently being the routine method for microanatomic examination of the tissue, requires extensive sample alteration by fixation, mechanical sectioning, and subsequent staining. Histology is therefore destructive, time-consuming, and cumbersome for the assessment of *in vitro* formed tissues. Recent advancements in optical tissue clearing methodology enable the use of confocal laser scanning microscopy in thicker tissues and could potentially replace histological analysis.^3^^–^^5^ Although optical tissue clearing enables deeper optical penetration^3^ and can be used in combination with exogenous fluorophores^4^ or endogenous fluorophores in transgenic animals,^5^ it requires tissue fixation and is therefore not a suitable approach for live tissue imaging. Thus techniques enabling real-time, label-free observation of organ formation would be desirable. Real-time and noninvasive imaging would not only allow for immediate control of culturing conditions but also provide refined methodology to study, for example, pharmacodynamics and kinetics.

Epidermis, the outermost skin layer, provides the physical and chemical barrier between the external environment and the human body and is important in the context of percutaneous absorption and transdermal drug delivery. Epidermis is formed in a complex process of epidermal differentiation. Keratinocytes, the main epidermal components, proliferate in the basal layer and undergo terminal differentiation while migrating vertically toward the skin surface. During differentiation, keratinocytes exhibit morphological and metabolic changes. The cells lose the organelles and form hexagonally shaped flat cornified envelopes at the surface of the skin.^6^ During this process, the biochemical composition of keratinocytes changes, particularly in the context of keratin expression. Proliferating cells in the basal layer express keratin 5 (K5) and 14 (K14), while differentiating cells express keratin 1(K1) and 10 (K10).^7^

Modern *in vitro* skin research concentrates on obtaining epidermis through the 3-D culture of human keratinocytes at air–liquid interfaces, where the cells are grown on membranes enabling nutrient delivery from one side of the membrane and contact with air on the other.^8^^–^^10^ Such 3-D culturing can be achieved on an acellular matrix such as a polycarbonate membrane^10^^–^^12^ or a cellular matrix such as a collagen matrix populated with fibroblasts^13^ to mimic fibroblast–keratinocyte paracrine interactions in native human skin.^14^^,^^15^ As with other organ culture models, present techniques for assessing *in vitro* formed skin are primarily destructive, and noninvasive methodologies are therefore desired.

The main methods used for noninvasive, label-free dermatological evaluations, both clinically and experimentally, are optical coherence tomography (OCT),^16^ reflectance confocal microscopy (RCM),^17^ and multiphoton microscopy (MPM),^18^^,^^19^ all techniques operating in the near-infrared (NIR) regime. Although OCT gives a possibility for deep-tissue imaging and imaging of large surface areas, it lacks resolution at the cellular level.^16^ Both RCM and MPM offer cellular resolution and similar imaging depth in skin;^18^^,^^20^ however, the principles behind the techniques differ. The imaging contrast in RCM is primarily dependent on the variability of the refractive index,^20^ while MPM is based on visualizing fluorescence.^21^ When applied to noninvasive and label-free investigations of the skin, MPM is primarily based on two-photon excitation of intrinsic cellular fluorophores, such as nicotinamide adenine dinucleotide (NADH), flavin adenine dinucleotide (FAD), and keratin.^22^^–^^26^ Additional MPM modalities, such as fluorescence lifetime imaging (FLIM) or spectral detection,^27^^–^^29^ would enable further fluorophore separation. This can provide information regarding cellular metabolism^30^ to complement morphological data, making MPM a suitable noninvasive imaging technology to use in combination with *in vitro* tissue culturing. Although FLIM and spectral detection analysis are beyond the scope of the presented work, MPM has been chosen for imaging due to its multimodal capacities that are of future interest. Furthermore, photodamage in MPM is minimized both by operating fs-pulsed NIR laser light and because the two-photon excitation process is confined to the focal point.^31^ Recently, MPM has been used for tracking stem cell self-renewal during epidermal differentiation in mice.^32^ However, most of the MPM skin-related research so far focuses primarily on transdermal drug delivery^33^ and clinical applications,^19^ including cancer diagnostics.^34^^–^^36^

The aim of this project was to investigate the potential for using MPM to detect cellular autofluorescence to provide a label-free and noninvasive tool to monitor epidermal differentiation *in vitro*. Of particular interest was developing an approach to visualize changes in the structure and morphology of *in vitro* skin models under varying ${\mathrm{Ca}}^{2+}$ concentrations. Extracellular ${\mathrm{Ca}}^{2+}$ is widely accepted in the scientific community to trigger terminal differentiation *in vitro*.^10^^,^^37^^–^^40^ Thus in this work, we utilize the effect of varying the extracellular ${\mathrm{Ca}}^{2+}$ concentration to influence differentiation and assess MPM as a method to detect morphological changes in *in vitro* skin cultures.

## 2 Materials and Methods

### 2.1 Cell Culture

Neonatal human epidermal keratinocytes (HEKn, Thermo Fisher Scientific) were cultured in Epilife^®^ (Thermo Fisher Scientific) growth medium supplemented with 60  μM of ${\mathrm{Ca}}^{2+}$, 1% human keratinocytes growth supplement (HKGS, Thermo Fisher Scientific), and 0.2% gentamicin/amphotericin (G/A, Thermo Fisher Scientific). The addition of HKGS to the growth medium provides 0.2% bovine pituitary extract, 1  μg/mL recombinant human insulin-like growth factor-I, 0.18  μg/mL hydrocortisone, 5  μg/mL bovine transferrin, and 0.2  ng/mL human epidermal growth factor. HEKn cells were seeded at the density of $2.5\times 10^{3}\text{ cells}/{\mathrm{cm}}^{2}$ in T-25 flasks in 5 ml of supplemented growth medium and incubated in a humified cell culture incubator at 37°C in 5% ${\mathrm{CO}}_{2}$ and 95% air. The growth medium was changed every other day until the cells reached 50% confluency and everyday thereafter, and cells were subcultured at 80% confluence.

### 2.2 *In Vitro* Epidermis Culture

**Fig. 1:**
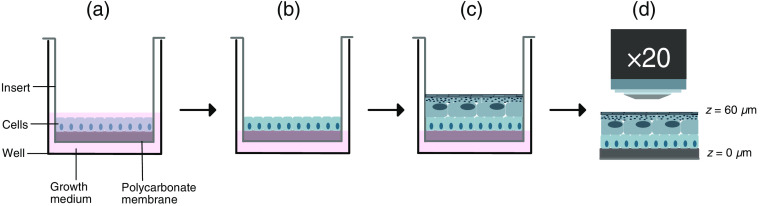
Schematic drawing of the experimental procedure: (a) HEKn cells are seeded on the polycarbonate membrane and cultured in regular growth medium for 24 h; (b) cells are exposed to the air–liquid interface by the aspiration of growth medium from above the cell culture, and the remaining medium is changed to a fully supplemented growth medium; (c) 3-D epidermal structure is formed after 11 days of cell culture; and (d) representative tissue cultures are cut out of the plastic insert and subjected to MPM investigation. The acquisition of the z-stack was initiated at the interface between the polycarbonate membrane and the intended basal cell layer (z=0  μm) and completed at the top of cornified layer (z≈60  μm, depending on the thickness of the model).

For 3-D tissue cultures, an established protocol for *in vitro* reconstruction of human epidermis at the air–liquid interface was implemented.^41^ The protocol was modified for the purpose of this study with respect to varying ${\mathrm{Ca}}^{2+}$ concentrations, the volume of growth medium, and the frequency of growth medium change. A schematic representation of the tissue culture protocol is shown in Fig. 1. The tissue cultures at varying ${\mathrm{Ca}}^{2+}$ concentrations were reproduced four times in this study. HEKn cells at passage numbers 2–5 were used, corresponding to 2–5 sequential subcultures since the culture initiation. Cells were seeded on polycarbonate filters with a 0.4-μm pore size and a surface area of $1.13\;{\mathrm{cm}}^{2}$ (Nunc™, Thermo Fisher Scientific) at the density of $2.5\times 10^{5}\text{ cells}/{\mathrm{cm}}^{2}$ and incubated in a 6-well plate with 2.5 ml of regular culture medium overnight in a humified cell culture incubator at 37°C in 5% ${\mathrm{CO}}_{2}$ and 95% air. After 24 h of incubation, the growth medium was replaced with 1 ml of fully supplemented Epilife^®^ growth medium: 1% HKGS, 0.2% G/A, 50  μg/ml vitamin C, and calcium with varying concentrations (0.00 mM, 0.03 mM, 0.30, and 3.00 mM). The cells were then exposed to the air–liquid interface by a gentle aspiration of the liquid above the membrane. The cells were then cultured for 14 to 21 days with a daily change of growth medium to allow for epidermis formation. Representative samples (N=4) from each ${\mathrm{Ca}}^{2+}$ concentration level were cut out of the plastic insert with a sharp scalpel and mounted as intact tissues on a microscope slide for MPM imaging.

### 2.3 Multiphoton Imaging

Imaging was performed on a multiphoton microscope LSM 710 NLO (Laser Scanning Microscope, Nonlinear Optical Imaging, Carl Zeiss, Jena, Germany). Two different 80-MHz fs-pulsed mode locked lasers were used for imaging: Mai Tai DeepSee (tunable in the wavelength region 700 to 1100 nm) and InSight DeepSee (tunable in the range of 680 to 1300 nm). All *in vitro* models were imaged with a 750-nm excitation. The laser power was manually increased for the deeper layers in the sample to acquire a visually comparable signal intensity from all z levels. Thus laser power varied in the range of 6 to 30 mW, as measured at the sample, depending on the depth of the imaging, with the lowest laser power being used at the surface of the sample. A water-immersion objective Plan Apochromat 20× (NA 1.0) was used, and autofluorescence in the range of 416 to 735 nm detected with a GaAsP detector. The z stacks were collected using ZEN Software (Carl Zeiss, Jena, Germany), scanning the x,y field of view (FOV) (425×425  μm, 1024×1024  pixels) using a pixel dwell time in a range of 0.79 to 1.58  μs, and a z step size of 1 to 2  μm. Tile imaging was performed by scanning up to 5×5 frames providing an FOV of up to 2125×2125  μm. Image processing was done using ImageJ^42^^,^^43^ (U.S. National Institutes of Health, Bethesda, Maryland) to enhance image contrast and increase the zoom factor.

## 3 Results

### 3.1 Tissue Architecture

**Fig. 2:**
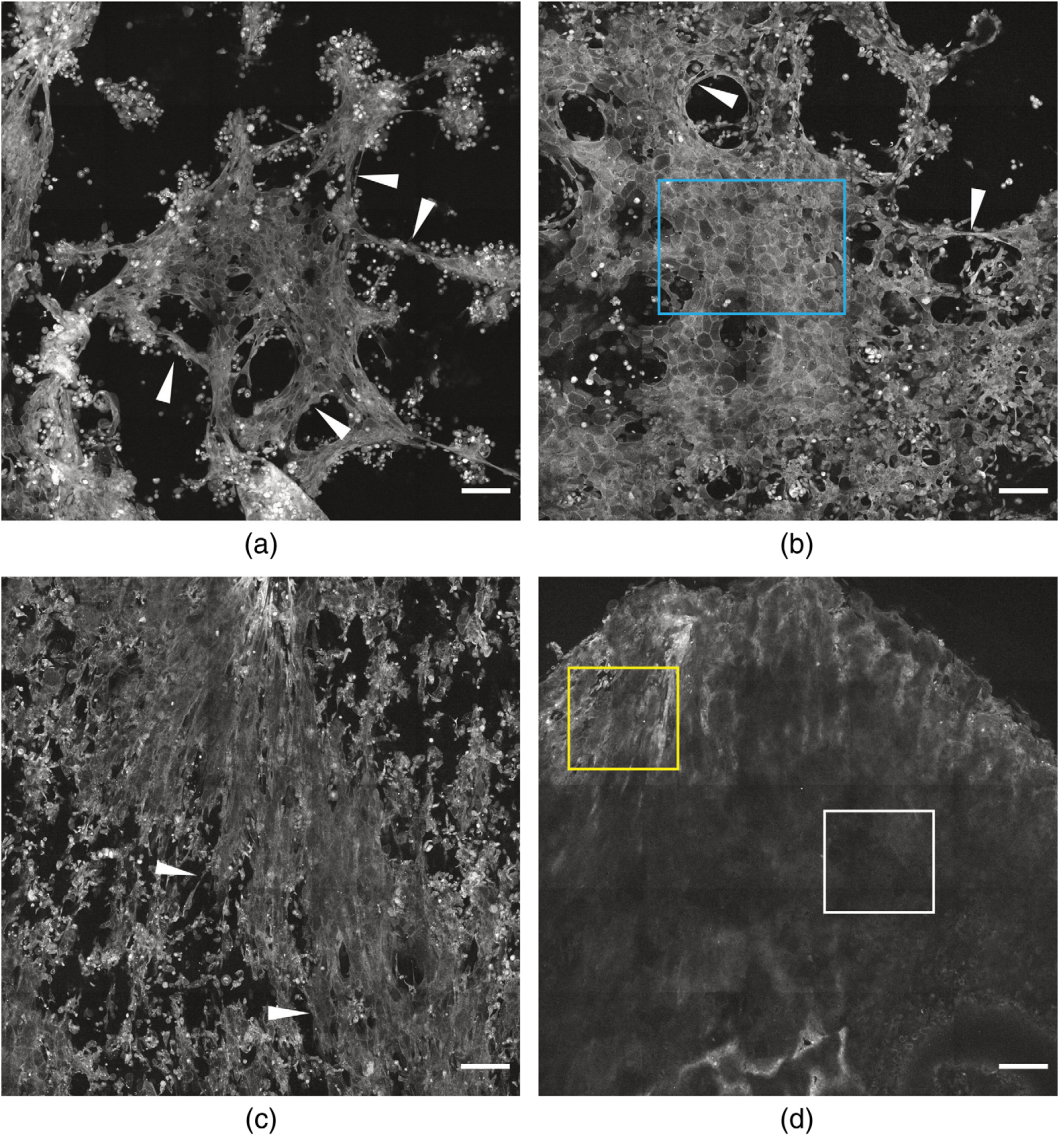
Large FOV autofluorescence MPM images (2125×2125  μm) of *in vitro* epidermal models cultured in growth media: (a) without ${\mathrm{Ca}}^{2+}$, (b) with 0.03 mM ${\mathrm{Ca}}^{2+}$, (c) with 0.30 mM ${\mathrm{Ca}}^{2+}$, and (d) with 3.00 mM ${\mathrm{Ca}}^{2+}$. The white arrows indicate the elongated cellular bridges. The blue ROI in (b) shows the sheet-like structure resembling a cornified layer of *in vivo* human skin. The yellow ROI in (d) shows the sheet-like structure resembling a cornified layer of *in vivo* human skin with high-fluorescent signal and the white ROI shows that with low-fluorescent signal. Scale bar is 200  μm. The contrast and brightness in the individual images have been adjusted for clarity.

Figure 2 shows representative MPM tile images, i.e., large FOV (2125×2125  μm), acquired from the tissue cultures grown in ${\mathrm{Ca}}^{2+}$ concentrations ranging from 0.00 to 3.00 mM. The tile imaging confirmed clear structural changes in the tissue architecture of the samples grown at different ${\mathrm{Ca}}^{2+}$ concentrations. Cells cultured without ${\mathrm{Ca}}^{2+}$ tended to grow in clusters [Fig. 2(a)], leading to uneven coverage of the polycarbonate membrane, and the cellular architecture was elongated. The cells grown in 0.03 mM ${\mathrm{Ca}}^{2+}$ [Fig. 2(b)] gave a higher membrane coverage, indicating a higher level of cell proliferation. At this ${\mathrm{Ca}}^{2+}$ concentration, a sheet-like structure resembling morphologically a cornified layer of *in vivo* human skin^44^ was observed with noticeable borders between the cells [highlighted by the blue region of interest (ROI) in Fig. 2(b)]. The cells cultured in 0.30 mM ${\mathrm{Ca}}^{2+}$ gave a more uniform coverage of the membrane [Fig. 2(c)], although some degree of clustering could be observed. Long intercellular bridges were observed at ${\mathrm{Ca}}^{2+}$ levels 0.00 to 0.30 mM [highlighted as white arrows, Figs. 2(a)–2(c)], suggesting the occurrence of intercellular communication. As expected, the cells grown in 3.00 mM ${\mathrm{Ca}}^{2+}$ formed the most tissue-like structure [Fig. 2(d)], as determined by a uniform and almost completely intact tissue architecture. The variation in fluorescence signal intensity across the sample is most probably dependent on changes in tissue topography during the optical sectioning [highlighted by yellow and white ROIs Fig. 2(d)]. This inherent effect becomes evident during tile scanning. Additional data (Fig. S1 in the Supplementary Material) suggest that epidermal differentiation might be influenced by cell density. Taken together, these results confirm that MPM enables visualization of changes in tissue architecture.

### 3.2 Morphology

**Fig. 3:**
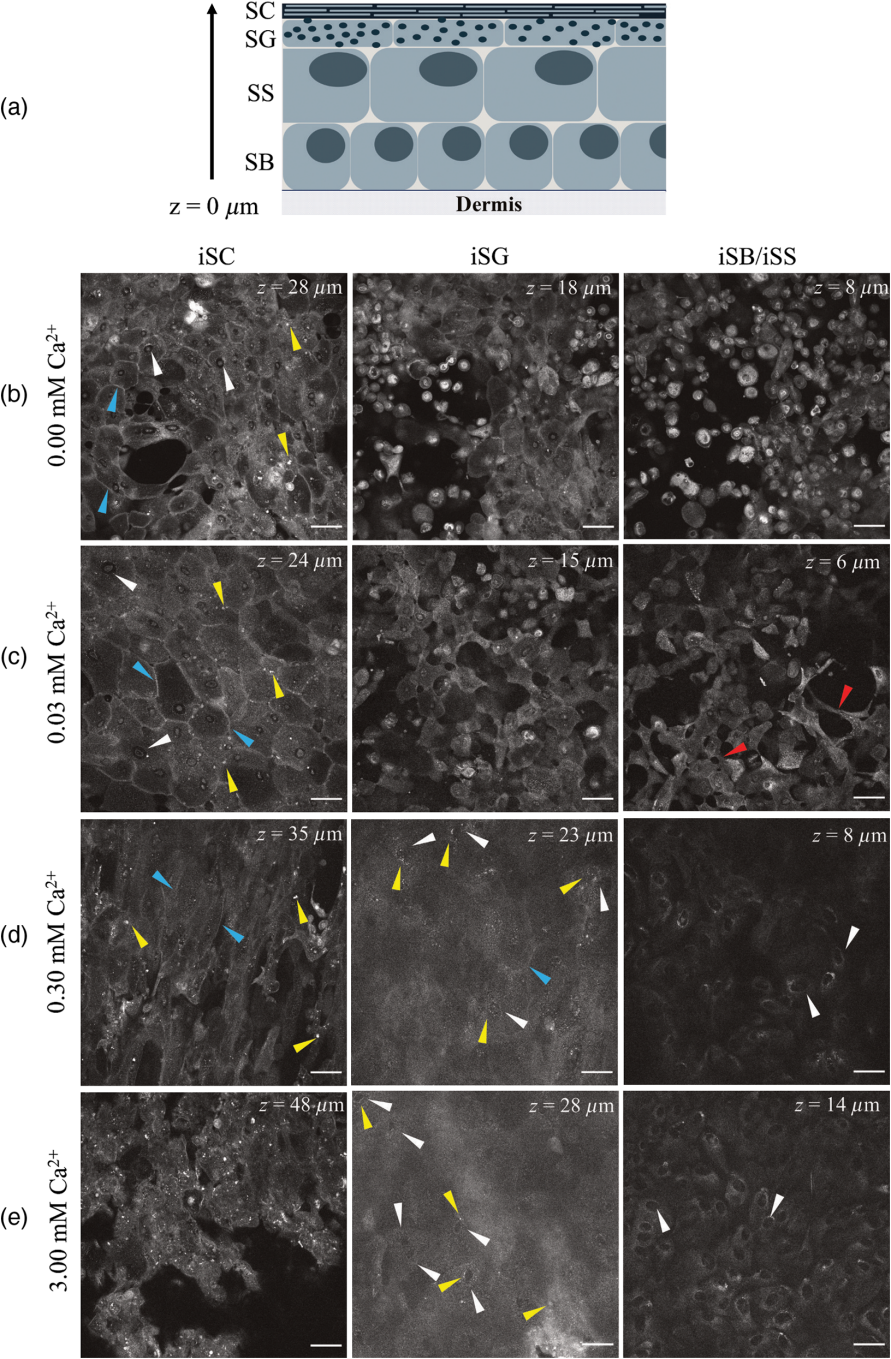
Autofluorescence MPM images corresponding to different strata of 3-D HEKn epidermal models *in vitro*. (a) Schematic drawing of epidermal strata for comparison (SC, stratum corneum; SG, stratum granulosum; SS, stratum spinosum; and SB, stratum basale). MPM images acquired from tissue models cultured in growth medium: (b) without ${\mathrm{Ca}}^{2+}$, (c) with 0.03 mM ${\mathrm{Ca}}^{2+}$, (d) with 0.30 mM ${\mathrm{Ca}}^{2+}$, and (e) with 3.00 mM ${\mathrm{Ca}}^{2+}$. The z values represent the distance of the z plane from the polycarbonate membrane, corresponding to the different intended strata (iSC, iSG, and iSB/iSS). Arrows represent: white, nuclei; yellow, granules; blue, cell boundaries; and red, cellular bridges. Scale bar is 50  μm. The contrast and brightness in the individual images have been adjusted for clarity.

Figure 3 shows individual MPM images acquired at different depths in four tissue samples cultured at different ${\mathrm{Ca}}^{2+}$ concentrations, together with a schematic representation of the epidermal differentiation process. Clear morphological differences in the cells grown at different ${\mathrm{Ca}}^{2+}$ concentrations could be identified with MPM (Fig. 3). The schematic drawing [Fig. 3(a)] illustrates the following expected epidermal strata: stratum basale (SB), stratum spinosum (SS), stratum granulosum (SG), and stratum corneum (SC). The MPM images were marked as intended strata (iSB, iSS, iSG, and iSC) based on the depth in the model and the observed morphology of the cells to facilitate the correlation of epidermal structure to autofluorescent features observed in native human skin.^44^ As expected, the cells formed distinct layers, which differed structurally, and exhibited morphological differences dependent on the ${\mathrm{Ca}}^{2+}$ concentration.

The outermost cellular layer formed in the models was defined as iSC. Cellular structures in the iSC differed significantly depending on the ${\mathrm{Ca}}^{2+}$ concentration. A thin layer of large flat cells with distinct cell boundaries (blue arrows) resembling the morphology of SC was found in low ${\mathrm{Ca}}^{2+}$ [≤0.03  mM, Figs. 3(b) and 3(c)]. However, visible nuclei (white arrows) and small round granules with high-fluorescent signal (yellow arrows) are expected to be found in SG, making the designation of that layer in the model questionable. Additional biochemical analysis is needed to confirm whether the cellular layers at these ${\mathrm{Ca}}^{2+}$ levels biologically correspond to SC. The tissue structure seen in iSC cultured at high ${\mathrm{Ca}}^{2+}$ [≥0.30  mM, Figs. 3(d) and 3(e)] was uniform, with visible cell boundaries (blue arrows) and without nuclei, consistent with SC in native tissue. However, granular structures (yellow arrows) were still visible.

In the iSG layer, the primary flattening of the cells (an event which later leads to iSC formation) could be observed in Figs. 3(b) and 3(c). In cells grown in 0.03 mM ${\mathrm{Ca}}^{2+}$, intercellular bridges formed. In comparison with MPM imaging of human skin,^44^ the iSG in Figs. 3(b) and 3(c) did not resemble the native tissue. However, the observed iSG layer in samples at high ${\mathrm{Ca}}^{2+}$ (≥0.03  mM) exhibited a low-fluorescent signal with a few nuclei (white arrows) surrounded by small fluorescent granules (yellow arrows). Additionally, a strong fluorescent signal was observed in Fig. 3(e) at z=28  μm originating from small round granules. The distinct cell–cell boarders and nuclei present in the additional samples correlated with the expected SG morphology. This might have been a consequence of the higher cell density observed in iSB/iSS (see also Fig. S3 in the Supplementary Material).

The iSB/iSS panels in Fig. 3 show the transition state between iSB and iSS. Cells that formed iSB/iSS without ${\mathrm{Ca}}^{2+}$ [Fig. 3(b)] stayed round with a high-fluorescent signal, suggesting that problem with cell adherence might be occurring.^45^ Cells cultured in 0.03 mM ${\mathrm{Ca}}^{2+}$ [Fig. 3(c)] grew densely and created intercellular bridges [Fig. 3(c), red arrows]. Basal cells observed in both 0.30 mM and 3.00 mM ${\mathrm{Ca}}^{2+}$ had large dark nuclei and fluorescent cytoplasm consistent with proper cell adhesion to the polycarbonate membrane^45^ [Figs. 3(d) and 3(e)]. No significant difference was observed between iSB/iSS in Figs. 3(d) and 3(e). (For complementary data and more detailed morphological analysis, refer to Figs. S2 and S3 in the Supplementary Material.)

## 4 Discussion and Conclusions

Here we demonstrate that MPM can be utilized as a label-free and noninvasive technique for the visualization and morphological classification of *in vitro* epidermal differentiation based on intrinsic tissue fluorescence. We employ MPM to detect ${\mathrm{Ca}}^{2+}$-induced differences in the tissue architecture and morphology of 3-D *in vitro* cell cultures. The tissue architecture was visualized with tile imaging, while the individual and zoom-in images provided detailed morphological information at the cellular level. The morphological features observed using MPM agree with histological analysis of *in vitro* cultured epidermis reported by others^41^ and resemble MPM images of intrinsic fluorophores from native human skin.^44^ Histology, currently being the gold standard for microanatomic examination of biological tissues, is destructive and requires extensive tissue processing before imaging. Thus the application of MPM as a label-free, 3-D, and potentially real-time approach to monitor tissue culturing is expected to play an important role in the future development of novel organ-on-a-chip models and advanced tissue engineering.

In this study, two-photon excitation using a 750-nm wavelength generated fluorescence from endogenous chromophores in a label-free manner. It is well known that the autofluorescence in the visible range using two-photon excitation primarily originates from NADH, FAD, and keratin.^22^^–^^26^ In this specific study, the visualization of ${\mathrm{Ca}}^{2+}$-induced morphological changes was of primary interest. It should be noted that similar morphological features could potentially be recognized with other optical modalities, e.g., OCT and RCM. For example, these techniques have already been used for noninvasive *in vivo* studies of tissue regeneration.^46^^,^^47^ Our rationale for choosing MPM was primarily based on its ability to visualize intrinsic tissue fluorescence, and future work will explore the possibility of combining the morphological data with spectral signatures and FLIM^27^^–^^30^ to shed further light on the metabolic aspects of ${\mathrm{Ca}}^{2+}$-induced *in vitro* epidermal differentiation in the context of *in vitro* tissue culturing.

The safety threshold for two-photon excitation imaging with regard to DNA damage and oxidative stress has previously been defined at around 7 mW.^48^ The average laser power at the sample in our study was in the range of 6 to 30 mW, depending on the imaging depth in the sample, suggesting some damage at the cellular level could occur. However, it should be noted that deep tissue two-photon excitation imaging at the level of 30 mW should not lead to structural damage^31^ and is considered safe for *in vivo* human applications. Therefore, repeated laser exposure during tissue culturing and phototoxic aspects should be taken into consideration and should preferably be monitored during further development of the approach.

There are a number of protocols and methods available for *in vitro* epidermis culture,^8^^,^^41^^,^^49^ but so far the tissue models lack hair follicles, sweat glands, and other skin components. Furthermore, *in vitro* skin models are reported to exhibit a reduced barrier function,^50^^,^^51^ limiting their applicability as model systems in pharmaceutical development. Thus, to develop more complex tissue models, more refined tissue culturing protocols are required, which elevates the need for real-time monitoring to allow for better control of tissue culturing. As demonstrated by this study, MPM can be used to distinguish distinct ${\mathrm{Ca}}^{2+}$-induced morphological differences. We, therefore, consider that such an approach will improve the investigation of *in vitro* epidermal differentiation in real time and thereby contribute to an overall better understanding of *in vitro* organogenesis.

The process of epidermal differentiation and cornification depends on many factors, such as cell density,^52^ presence of vitamin C,^53^ exposure to the air–liquid interface,^49^ keratinocyte-fibroblast paracrine interaction,^14^ or extracellular ${\mathrm{Ca}}^{2+}$ levels.^39^ It is known that intracellular ${\mathrm{Ca}}^{2+}$ plays an important role in tissue homeostasis and desmosomal structure dynamics after barrier disruption,^54^ and cytoplasmic ${\mathrm{Ca}}^{2+}$ elevates just before cornification.^55^ Additionally, in the case of *in vitro* epidermis formation, the natural ${\mathrm{Ca}}^{2+}$ gradient in human skin should be considered: the peak concentration of ${\mathrm{Ca}}^{2+}$ levels is observed in SG, with a rather high concentration in SB and SS as well, that declines again in SC.^56^ Therefore, in this study, the cells cultured in low ${\mathrm{Ca}}^{2+}$ might have committed directly to the formation of SC without going through the earlier phases of differentiation. Additional tissue cultures with a lack of SC formation (Supplementary Material) support the interpretation that cell density is of importance in epidermal differentiation. It is plausible that those two factors, low extracellular ${\mathrm{Ca}}^{2+}$ and high cell density, lead to cornification. To confirm this, further studies focusing on the expression of differentiation markers (K5/K14 and K1/K10) are needed. It has previously been reported that a peak of cytoplasmic ${\mathrm{Ca}}^{2+}$ levels is observed immediately before cornification.^55^ It is, therefore, possible that the constant extracellular ${\mathrm{Ca}}^{2+}$ level in the *in vitro* tissue culture could influence the required decline in intracellular ${\mathrm{Ca}}^{2+}$, thereby affecting cornification. These aspects together further support the need for a noninvasive visualization tool to enable real-time monitoring of tissue formation and ${\mathrm{Ca}}^{2+}$-induced effects.

To conclude, we show that intrinsic tissue fluorescence visualized with MPM enables detailed 3-D imaging of morphological and structural features associated with epidermal differentiation *in vitro*. The expected epidermal layers present in human skin could be observed in the cultured models, and ${\mathrm{Ca}}^{2+}$-induced effects were clearly discerned. It should be noted that the *in vitro* skin culture protocols can be further refined to produce cultures fully resembling native human tissue.^57^^,^^58^ It is plausible that high cell density and the real-time control of ${\mathrm{Ca}}^{2+}$ gradient are, among others, important parameters needed for the development of improved *in vitro* tissue culturing protocols. For this purpose, MPM provides a feasible approach, potentially enabling real-time tissue culture optimization. Furthermore, MPM in combination with FLIM and spectral detection is expected to add important additional information in order to aid in the distinction of cell differentiation and proliferation. This approach is of interest not only for epidermal differentiation studies but also for any potential organ formation applications, such as personalized drug delivery and transplantation. Thus multimodal autofluorescence MPM is expected to become a significant technology that ultimately enables real-time, noninvasive, and label-free studies of tissue regeneration and organ formation *in vitro* as well as *in vivo*.
