# Supplementary material for: Monitoring calcium-induced epidermal differentiation in vitro using multiphoton microscopy
Source: J Biomed Opt. 2020 May 9;25(7):071205. doi: 10.1117/1.JBO.25.7.071205 (PMC7210787; doi:10.1117/1.JBO.25.7.071205)
Supplement: Supplementary file 1 [file JBO_025_071205_SD001.pdf]

Supplementary Material

Monitoring calcium-induced epidermal differentiation *in vitro* using multiphoton microscopy

Monika Malak,[a,*] Julie Grantham,[b] Marica B. Ericson[a,*]

[a] University of Gothenburg, Faculty of Science, Department of Chemistry and Molecular Biology, Biomedical photonics, Kemivägen 10, Gothenburg, Sweden, 412 96

[b] University of Gothenburg, Faculty of Science, Department of Chemistry and Molecular Biology, Medicinaregatan 9C, Gothenburg, Sweden, 413 90

Supplementary data

Figure S1 shows tile imaging of tissue architecture in *in vitro* epidermal models in the repeated experiment complementary to data in Figure 2 (main manuscript). Analogous tissue architecture was observed in the remaining samples (N=2) therefore, only one set of data is presented. In comparison to Figure 2, the MPM images here demonstrated a more homogenous cell growth. The cells grown in 0.03 mM Ca^{2+} seemed to have a higher proliferative rate in comparison to the cells grown without Ca^{2+}. The cells did not form the cornified-like layer as in Figure 2, suggesting that the physical cornification might be cell density dependent. The cells in the models grown in higher Ca^{2+} concentration formed a uniform sheet-like structure suggesting that the cornified layer was achieved. The high fluorescent signal observed in Fig. S1(c) and the yellow ROI in Fig. S1(d) would suggest that the cells were metabolically active at the time of imaging. The lack of signal in the white ROI in Fig. S1(d) is most probably correlated to the change in tissue topography, as seen in Fig. 2 (main manuscript).

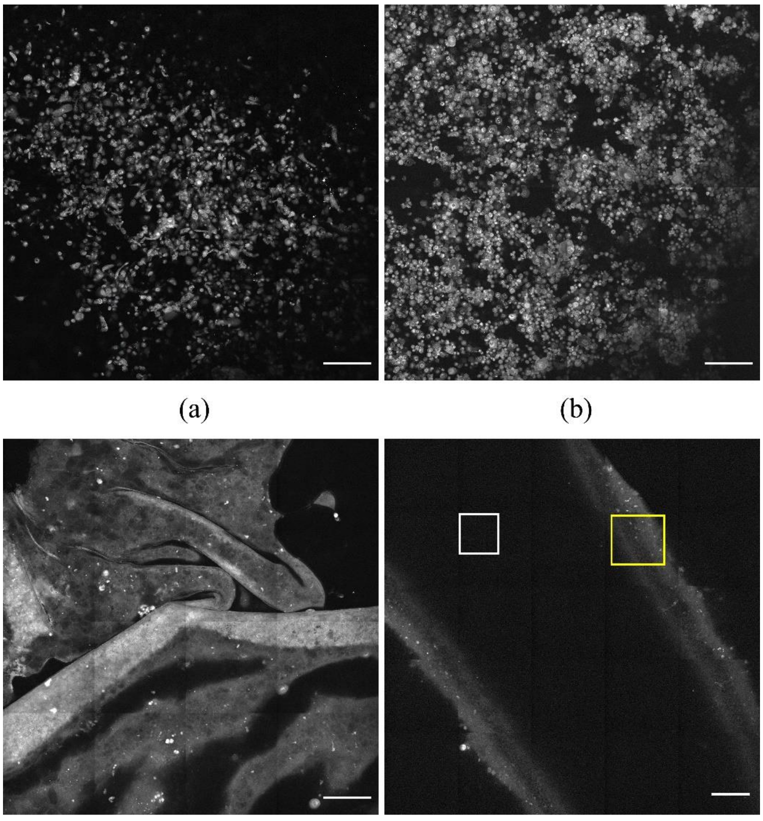

(a) (b)

(c) (d)

Fig. S1 Large FOV autofluorescence MPM images (1572 x 1572 µm) of *in vitro* epidermal models cultured in growth medium: (a) without Ca^{2+}, (b) with 0.03 mM Ca^{2+}, (c) with 0.30 mM Ca^{2+} and (d) with 3.00 mM Ca^{2+},

from a second tissue culturing batch complementary to the experiment reported in Figure 2 (main manuscript). The yellow ROI in (d) shows the sheet-like structure with high fluorescent signal and the white ROI, with low fluorescent signal. Scale bar 200 µm. The contrast and brightness have been adjusted for clarity.

Figure S2 shows autofluorescence MPM imaging of the cell morphology in *in vitro* epidermal models in the repeated experiment complementary to data in Figure 3 (main manuscript). Analogous morphology was observed in the remaining samples (N=2) therefore, only one set of data is presented. As seen in the figure, HEKn cells grown without Ca^{2+} formed one layer of rounded cells and did not form intercellular bridges or cell boundaries. The cells did not form a SC layer with nucleated flat cells with nonfluorescent nuclei in comparison to the observations discussed in Figure 3. Cell density might be the main factor leading to cornification.

In comparison to Figure 3, an improved morphology of iSB/iSS layer was observed in the model grown in 3.00 mM Ca^{2+} (Fig. S2(e)), which resembled SB and SS layers in native human skin[44]. Denser iSB/iSS layer might have led to the proper iSG formation with observed cell boundaries and nuclei. Large fluorescent particles in iSC and iSG in Fig. S2(e) might have been cell debris associated with high cell density.

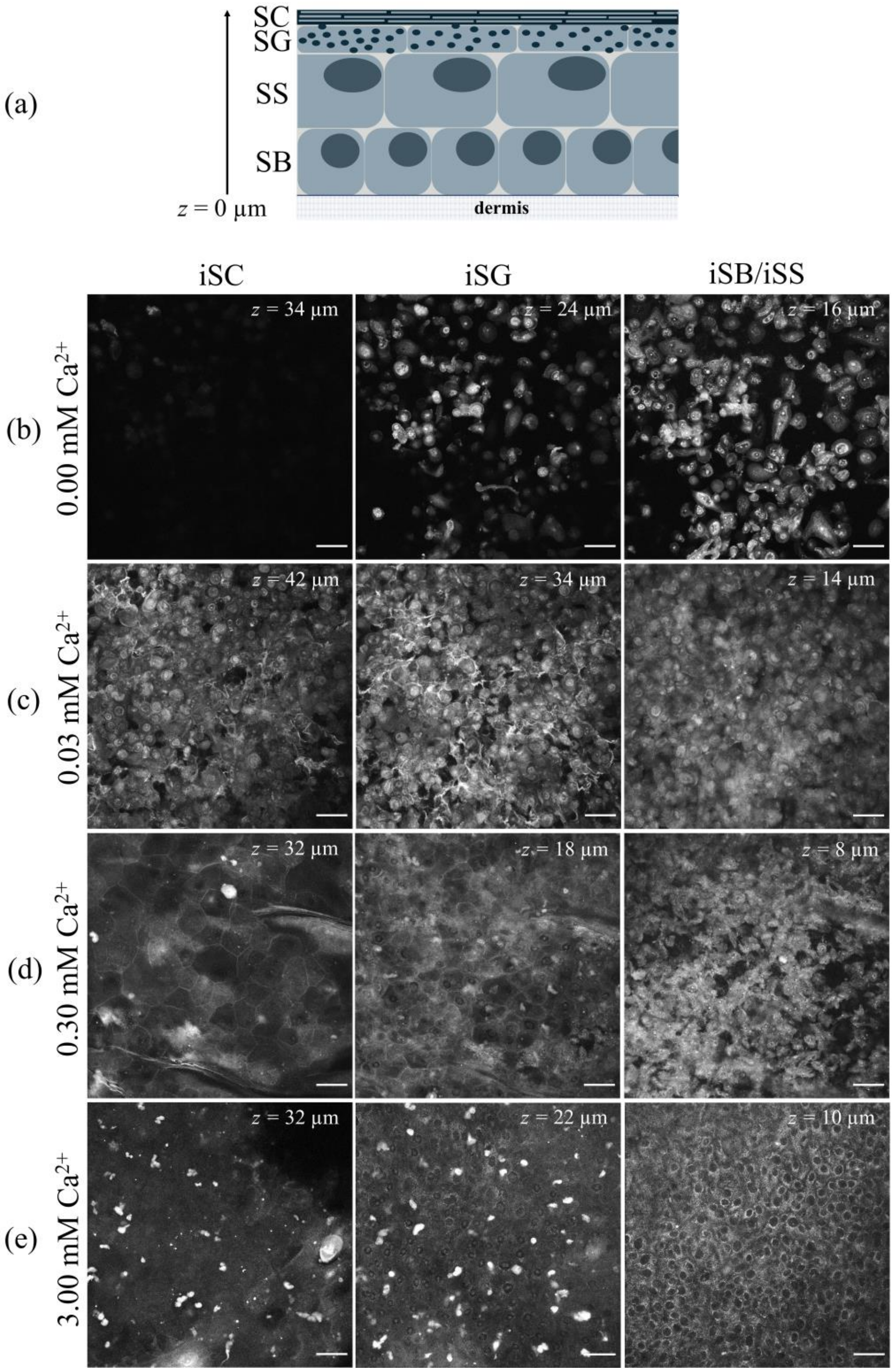

Fig. S2 Autofluorescence MPM images corresponding to different strata of 3D HEKn epidermal models *in vitro* from a second tissue culturing batch complementary to experiment reported in Figure3 (main manuscript). (a) Schematic drawing of epidermal strata for comparison. MPM images acquired from tissue models cultured in growth medium: (b) without Ca^{2+}, (c) with 0.03 mM Ca^{2+}, (d) with 0.30 mM Ca^{2+} and (e) with 3.00 mM Ca^{2+}. The z-values represent the distance of the *z*-plane from the polycarbonate membrane, corresponding to the

different intended strata (iSC, iSG, iSB/iSS). Note that no iSC was formed in 0.00 mM and 0.03 mM Ca^{2+}. Scale bar 50 µm. The contrast and brightness have been adjusted for clarity.

Figure S3 presents a zoom-in on morphological cellular features of the formed layers presented in Figure 3 (main manuscript). Some nuclei could be distinguished in the iSB/iSS (white arrows in Fig.S3(a,b)) at lower Ca^{2+} levels. Towards the surface, at z = 18 µm, the cells started to form a sheet-like structure through flattening and cell boundaries formation (blue arrows). At z = 28 µm dark nuclei could be observed with a surrounding void (white arrows), bright round structures suspected to be lipid droplets (yellow arrows) and formed cell boundaries (blue arrows).

Cells observed in iSB/iSS in Fig. S3(b) had a more elongated shape than the cells grown without Ca^{2+}. At z = 6 µm the cells grew more densely, and the nuclei were difficult to observe (white arrows). At z = 15 µm flattening and spreading of the cells could be already observed that led to the formation of uniform and intact sheet of flat and large cells. Some dark nuclei with a surrounding void were observed (white arrows) in the plane, as well as granular structures (yellow arrows), and cell boundaries (blue arrows).

Basal cells recognized in Fig. S3(c) were larger than in Fig. S3(a) and Fig. S3(b). Dark nuclei with a fluorescent cytoplasm suggest that the cells adhered properly to the membrane. Towards the surface, at z = 23 µm, the cells created a sheet-like structure with no distinguished borders. The observed white round bright particles (yellow arrows in Fig.S3) were most likely lamellar bodies, which are secreted to the extracellular space in SG during the differentiation in native tissue. The cells observed at z = 35 µm formed a sheet-like structure which seemed to be elongated on one axis. The distinct boarders between the cells (blue arrows) and large white granules (yellow arrows) were observed. No nuclei could be found.

Figure S3(d) shows the model grown in 3 mM Ca^{2+}. As in Fig. S3(c), dark large nuclei were observed with a bright signal originating from the cytoplasm, suggesting a proper cell adhesion to the membrane[43]. The signal arising at z = 28 µm was weak with similar granules as in Fig. S3(c). In the plane z = 48 µm, some nuclei were observed, and the cells formed a sheet-like structure without distinct borders.

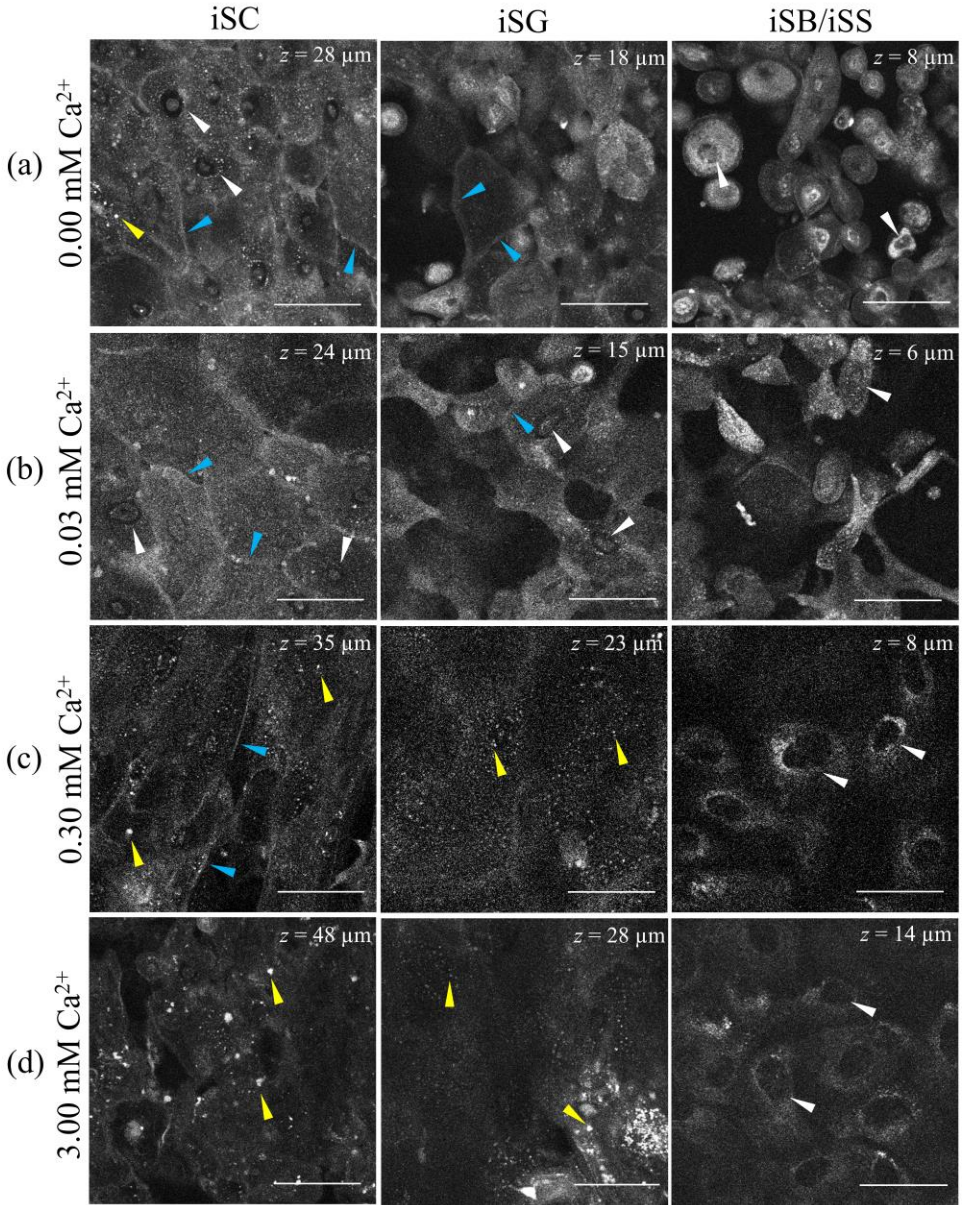

Fig. S3 Autofluorescence MPM images after digital zoom of data presented in Figure 3 (main manuscript) illustrating detailed cell morphology. Cells cultured in growth medium: (a) without Ca^{2+}, (b) with 0.03 mM Ca^{2+}, (c) with 0.30 mM Ca^{2+} and (d) with 3.00 mM Ca^{2+}. The *z*-values in the right upper corner represent the distance of the *z*-plane from the polycarbonate membrane. Arrows represent: white – nuclei, yellow – granular particles, blue – cell boundaries. Scale bar 50 µm. The contrast and brightness have been adjusted for clarity.
